# The effects of low levels of trivalent ions on a standard strain of *Escherichia coli* (ATCC 11775) in aqueous solutions

**DOI:** 10.1002/mbo3.574

**Published:** 2018-01-15

**Authors:** Can Deng, Xinpeng Li, Xinkai Xue, Richard M. Pashley

**Affiliations:** ^1^ POWERCHINA Water Environment Governance Shenzhen China; ^2^ School of Physical Environmental & Mathematical Sciences Canberra Australia

**Keywords:** *E*. *coli*, lanthanum, membrane failure, phospholipid vesicles, size distribution, trivalent cation

## Abstract

Considering the ever‐growing usage of trivalent salts in water treatment, for example, lanthanum salts in rare earth, AlCl_3_ and FeCl_3_, the effects of different trivalent cations on the bacterium *Escherichia coli* (*E. coli*) ATCC 11775 strain have been studied in aqueous solutions. From colony incubation studies, the colony‐forming unit (CFU) densities were found to decrease significantly in the presence of even low levels (10^−5^ mol/L) of lanthanum chloride. This level of reduction in CFU number is comparable to the results obtained using the known bacteriocidal cationic surfactant, C_14_ TAB. By comparison, exposure of the cells to low levels of trivalent ion, aluminum and chromium ion solutions produced only modest reductions in CFU density. The results from the incubation studies suggest that the bacteriostatic mechanism of La^3+^ ions has similarities to that of the cationic surfactant, and different to that of the other trivalent ions. Size distribution and zeta potential measurements of *E*. *coli* cells and phospholipid vesicles in the presence of trivalent cations solutions suggested significant cell shrinkage probably caused by membrane disruption.

## 1 INTRODUCTION

Escherichia coliform (*E*. *coli*) cells are standard indicator organisms for industrial water purification and wastewater treatment systems worldwide. Specifically, they are able to indicate possible sewage contamination, possible presence or absence of pathogenic bacteria, viruses and protozoa in water (Kay et al., 2008; USEPA 2012). A thorough understanding of surface characteristics of this microorganism in different aqueous solutions is therefore important for the detailed analysis of comparative water treatment processes and their further development.

A detailed understanding of the surface electrostatic charging properties of *E*. *coli* cells in electrolyte solutions can be used to analyze the mechanisms involved in many physicochemical water treatment processes. The cell surface charging properties of the *E*. *coli* strain ATCC 11775 was analyzed in monovalent and divalent salt solutions of NaCl and CaCl_2_ in a previous study (Li, Xue, & Pashley, 2015). In this paper, the effect of trivalent cations has been examined. It is of interest to study the effects of different multivalent ions on cells. These extend and complement specific ion Hofmeister effects, on bacterial growth (Pierandrea Lo et al., 2005). Multivalent salts can often be used at much lower concentrations than monovalent salts for reducing and screening surface electrostatic effects in water treatment processes.

Trivalent salts like AlCl_3_ and FeCl_3_ are used as flocculants in water treatment processes (Han, Runnells, Zimbron, & Wickramasinghe, 2002; Pouet & Grasmick, 1995; Sholkovitz, 1976). Two characteristics of trivalent cations account for their widespread use, in neutralizing negatively charged surface of particles by the adsorption of Fe^3+^ and Al^3+^ ions and their hydrolyzed forms, such as Fe(OH)^2+^, Al(OH)^2+^ (Duan & Gregory, 2003) and in “sweep flocculation” to encapsulate particles in the form of extensive amorphous hydroxide precipitates (Bulson, Johnstone, Gibbons, & Funk, 1984; Duan & Gregory, 2003). The effectiveness of these mechanisms depends on highly variable factors such as the solution conditions, presence of competing ions, as well as salt concentration and pH.

Thus, the electrostatic effects of trivalent cation adsorbed onto negatively charged coliform surfaces are difficult to study separately from those of the divalent and monovalent cations produced via ion hydrolysis. Furthermore, a significant pH reduction caused by iron or aluminum hydrolysis will significantly change the viability of *E*. *coli* cells. It would seem to be impossible to obtain single component trivalent Al^3+^ and Fe^3+^ cations in the electrolyte solutions of interest in water treatment.

Nonetheless, knowledge of the behavior of these ions is important because of their direct effects on bacterial cells. Fe^3+^ in concentrations of about 10^−3^ mol/L has been shown to cause a slight decrease in *E*. *coli* growth (Kalantari & Ghaffari, 2008). In contrast to Fe^3+^, Al^3+^ shows significant inhibition effects on *E*. *coli* cell growth, at the similar concentration of 9 × 10^−4^ mol/L at a pH value of 5.4 (Guida, Saidi, Hughes, & Poole, 1991). It has been reported that the toxicity of Al^3+^ to *E*. *coli* increased as the pH was decreased (for pH value < 5.4) (Guida et al., 1991). This indicated that the Al^3+^ ion was responsible for toxicity in these aqueous solutions (Guida et al., 1991; Piña and Cervantes 1996).

By comparison, chromium (III) and lanthanum salts are less readily hydrolyzed and only minimal pH changes are produced in dilute solutions (Hu, Chandran, Smets, & Grasso, 2002). However, chromium has been reported to have inhibitory effects on the growth of bacteria (Kalantari & Ghaffari, 2008; Yao et al., 2008). This suggests that lanthanum salts might be the most useful for the study of effects that can safely be attributed entirely to the highly charged cation, with minimal associated pH changes.

There are contradictory reports on whether low concentrations of the lanthanum cation might or might not inhibit microorganism growth. Zhang et al. (2010) and Peng, Hongyu, Xi, Chaocan, and Yi (2006) reported that the low concentration of, down to 4 × 10^−4^ mol/L, lanthanum cation increased the nutritional uptake of *E*. *coli* cells and did not produce any toxic effect. Wenhua, Ruming, Zhixiong, Xiangdong, and Ping (2003) claimed that La^3+^ at concentrations from 2 to 6 × 10^−4^ mol/L stimulated the cell metabolism. By comparison, Burkes & McCleskey, 1947), report that *E*. *coli* growth can be affected by adding 2 × 10^−4^ mol/L lanthanum chloride. In addition, Wenhua et al. (2003) and Rim, Koo, and Park (2013) showed that La^3+^ at the concentration range 0.02–1.2 × 10^−4^ mol/L effectively inhibited *E*. *coli* from absorbing external DNA, decreasing the transformation efficiency. Since each of these experiments used different growth media, different cell strains and densities, controversial conclusions can be drawn from these studies. Therefore, our focus here is to determine whether low concentrations of the lanthanum salt can have an effect on *E*. *coli* bacterial growth or not based on the study of incubation and surface properties.

## 2 MATERIALS AND METHODS

### 2.1 Solutions preparation

Certified reagent‐grade chemicals (≥99% purity), HEPES powder, sodium hydroxide (NaOH), sodium chloride (NaCl), and myristyltrimethylammonium bromide (C_14_TAB) were supplied by Sigma‐Aldrich, while aluminum chloride hexahydrate (AlCl_3_·6H_2_O) and chromium chloride hexahydrate (CrCl_3_·6H_2_O) were both from Fluka Analytical. All chemicals were used without further purification; 17.9‐mmol/L ferric chloride (FeCl_3_) standard solution and 26.6% w/v lanthanum chloride (LaCl_3_) solution were obtained from BDH Chemicals. Aqueous solutions were prepared by deionized, ultrafiltered water (Milli‐Q). At room temperature, the deionized water had a conductivity <2.0 μS/cm and a natural equilibrium pH of 5.7.

The strain of *Escherichia coli* (*E*. *coli*) used in this study was ATCC 11775, due to its potential application as a monitor organism for general water treatment processes. It is also a nonpathogenic strain (Eblen, Annous, & Sapers, 2005) and so can be used with “Biosafety Level 1” procedures. The surface charging properties of this strain in 10^−3^ mol/L NaCl solution with varied pH value (3–9) has been studied and the results showed that it had a negative surface charge, as expected (Li et al., 2015).

All cell suspension solutions of around 10^8^ cell/ml in this study were purchased from ALS Environmental Nowra Laboratory, NSW. The coliform densities were also remeasured before each run of the experiment. The results showed that the cell density typically varied with time and in the range of 2 × 10^8^–5 × 10^8^ CFU/ml, over a period of about a week. It should be noted that all the cell suspension samples were only used within 1 week. This suspension solution initially contained 1.0 mg/L Tryptone (a defined peptone) from Thermofisher Scientific and had a pH value of about 7.2 at room temperature. In all experiments, the suspension solution was substantially diluted, typically by 100× or more, to minimize the effect of culture medium on membrane filtration (MF) and size measurements.

### 2.2 Coliform density evaluation method

The membrane filtration (MF) (Baker, 2012; Porter, 1989) technique was used to estimate coliform bacteria populations in the solutions used. In each experiment, a required amount of water sample was collected from well‐mixing solution, using a sterile syringe. Typically these samples were diluted with sterilized water to optimize the fecal coliform count. The samples were filtered through sterile 47 mm gridded membrane filters made from mixed esters of cellulose with 0.45 μm pore size. In the case of ATCC 11775 strain *E*. *coli* study, the filters were placed in petri dishes containing m‐ColiBule24 broth and incubated for a period of 24 ± 2 hr at 37°C. The broth is designed for growth and detection of total coliform and *E*. *coli*. The growth media were obtained from Millipore Corporation. Following incubation, the petri dishes were removed from the incubator and coliform colonies were counted in each dish. The average coliform density of each sample is from three MF evaluation results.

The ideal sample volume of solution for coliform testing should be in the range between 0 and 100 CFU per filter, either because the numbers are too low or too high, where individual colonies would inevitably become combined. Therefore, the suspension solution of the coliform cell was substantially diluted, typically by 5 × 10^6^, using 10^−3^ mol/L NaCl sterilized water, in order to gain the required coliform density solution in the MF. For the coliform viability study, the same diluted solution was studied. Around 1 ml, 2 ml, or 100 ml of sample was analyzed using the MF procedure. The diluted cell suspension solution only used within 24 hr, because the reproducibility within samples was good, but sample to sample reproducibility was variable.

### 2.3 Phospholipid vesicles preparation

L‐α‐phosphatidylserine (PS) powder (≥97% purity) and L‐α‐phosphatidylcholine (PC) powder (≥99% purity) were purchased from Sigma‐Aldrich. Small unilamellar vesicles (SUVs) of the phospholipids were prepared by sonication using the method of Morrissey (2001). PS and PC were solubilized in HEPES/NaOH buffer solution (0.02 mol/L HEPES, 0.1 mol/L NaCl, adjusted to a pH 7.5 using NaOH) at room temperature by a molar ratio of 20:80, to give 1 mg/ml phospholipids (PL). The solution was then vortexed vigorously to obtain a milky uniform suspension. Using a Branson 5,200 bath sonicator, the suspension solution was sonicated at room temperature for around 20 min and the appearance of the solution turned quite clear. The final vesicle solutions were stored at 4°C and used within 48 hr after preparation.

It has been established that the size of the SUVs should be around 100 nm (Mui, Chow, & Hope, 2003) and so 220‐nm‐diameter pore polycarbonate filters, obtained from Merck Millipore, were used to filter the SUV solution at low temperature. At low temperatures, the fairly rigid SUVs are more easily filtered out.

Dynamic light scattering (DLS) measurements were performed within hours after sonication. For vesicle size and zeta potential measurements, each sample was diluted to a final concentration of 0.05 mg/ml, using the HEPES/NaOH buffer in which the phospholipid was initially suspended.

### 2.4 Size distribution and zeta potential measurement

Size distribution of the standard *E*. *coli* cells and the SUVs were determined using a Nano Zeta Sizer (Zetasizer Nano ZS Malvern Instruments Ltd). This zeta sizer was also used for zeta potential measurements of the SUVs. The zeta potential of each sample was measured five times to ensure the accuracy of the results.

For the DLS measurement of the Zeta Sizer, the refractive index (RI) of *E*. *coli* cells was 1.395 (Balaev, Dvoretski, & Doubrovski, 2002, 2003), and that of the membrane lipids should be around 1.48 (Chong & Colbow, 1976). RI of these materials are needed only to change the distribution from intensity based to a volume or number based, and so in intensity measurements, they are not critical (Malvern, 2009). Unlike the spherical shape of SUVs, *E*. *coli* cells have a basic rod‐shape. The cell size was measured in terms of the effective hydrodynamic diameter of the *E*. *coli* cell. Therefore, the reported values correspond to an equivalent spherical diameter. It should be noted that, for those samples with polydispersity index (PDI) values over 0.5, having a broader distribution, the peak value is more acceptable than the *Z*‐average (number average) in size distribution analysis (Malvern, 2009). For low‐density cell solution measurements, forward scattering was chosen as an acceptable standard model, as the forward angle data contain more signals from larger particles present in the diluted solutions (Nobbmann, 2014).

Electrical conductivities and pH values of solution samples were measured using a EUTECH PC700 instrument. A Millipore single chamber incubator was used to process the petri dishes containing the cells.

## 3 RESULTS

### 3.1 ATCC 11775 strain *E*. *coli* inactivation in low concentration lanthanum chloride solutions

**Figure 1:**
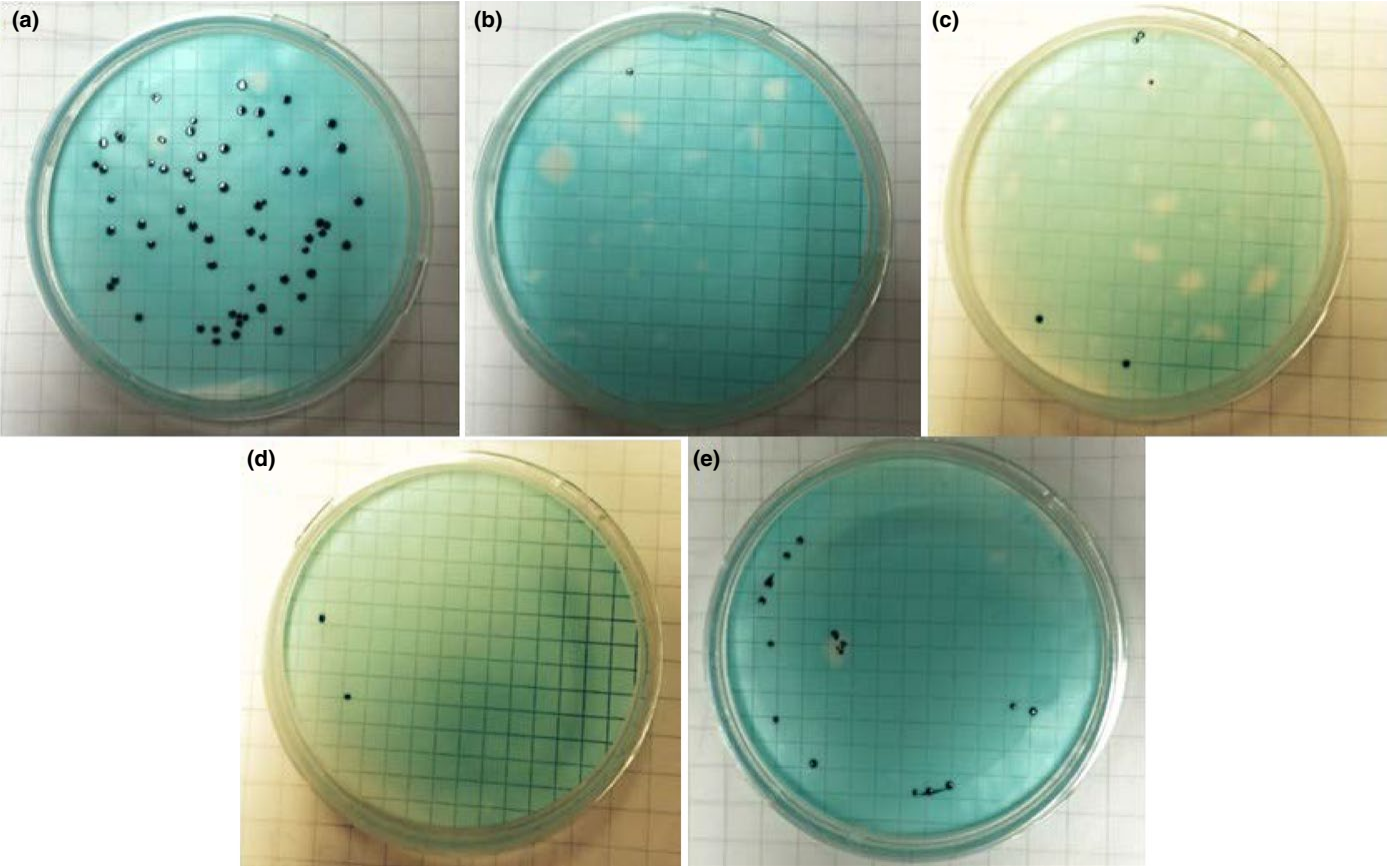
Photographs of the coliform colonies obtained from defined volume samples of 50 CFU/ml density ATCC 11775 strain *Escherichia coli* diluted suspension solutions with 10^−3^ mol/L NaCl, with and without LaCl_3_, following membrane filtration process, and then 24 hr of incubation at 37°C with m‐ColiBlue24 broth. (a) 1 ml of the suspension solution without LaCl_3_ (b) 1 ml of the suspension solution with 10^−5^ mol/L LaCl_3_ (c) 100 ml of the suspension solution with 10^−5^ mol/L LaCl_3_ (d) 1 ml of the suspension solution with 10^−6^ mol/L LaCl_3_ (e) 100 ml of the suspension solution with 10^−6^ mol/L LaCl_3_

Initially, a study was carried out on the effect of low concentrations of added lanthanum chloride on coliform colonies via incubation. The photos in Figure 1 showed the effect on coliform inactivation, with and without added lanthanum. To obtain reasonable counting, each plate had a density of around 50 CFU/ml diluted *E*. *coli* cell suspension solution 1 ml, as approved by Figure 1a, that is, the blank group of *E*. *coli* sample without lanthanum chloride. By comparison, the results in Figure 1b and d gives the results for 10^−5^ mol/L and even lower 10^−6^ mol/L lanthanum cation added into the solutions. Clearly, the lanthanum ions had a significant inhibition effect on coliform growth. Furthermore, only a few colonies were observed in Figure 1c and e, using the 100 ml samples with 10^−5^ mol/L and 10^−6^ mol/L lanthanum chloride, respectively. These results clearly demonstrated that the low concentration added lanthanum in the solutions can inactivate this standard *E*. *coli* strain and affect the coliform colonies growing.

### 3.2 Comparison of lanthanum ions with other trivalent ions on *E*. *coli* viability

The MF technique can fail to distinguish between single cell and an aggregate group of cells, and both of which can produce one colony on the growth plate. Thus, it is difficult to identify the differentiation between single *E*. *coli* cells and aggregated cells. A similar aggregation phenomenon has also been reported in a study of viruses (Brennecke, 2009). Hence, it is important to distinguish the colony reductions with simple cell aggregation, which might have been caused by the presence of low level of lanthanum ions.

As already remarked, trivalent salts, for example, AlCl_3_ and FeCl_3_ are widely used as coagulants in water and wastewater treatment; they are effective in removing a broad range of impurities from water, including colloidal particles and dissolved organic substances (Bulson et al. 1984; Duan & Gregory, 2003; de Godos et al., 2011), generally because of their formation of a range of hydrolyzed species in solution. To avoid effects of sweep flocculation (Duan & Gregory, 2003), and exclude the inhibiting growth effects of AlCl_3_ and FeCl_3_ at around 10^−3^ mol/L (Guida et al., 1991; Kalantari & Ghaffari, 2008), small dosages of 10^‐5^ mol/L AlCl_3_, FeCl_3_, and LaCl_3_ were added to the diluted standard *E*. *coli* solutions with 50 CFU/ml initial coliform density, 2 ml for direct comparison.

The results obtained are summarized in Table 1, which showed the effects of these three trivalent cations on *E*. *coli* cell incubation. The results clearly demonstrated that there was almost the same level of reduction in Al^3+^ and Fe^3+^ solutions in terms of CFU, from 100 CFU per plate to 50–60 CFU per plate. This was most likely due to charge neutralization and cell aggregation. However, unlike Al^3+^ and Fe^3+^, the presence of added La^3+^ produces a substantial reduction in colony numbers, from 100 CFU per plate to 2 CFU per plate. By comparison, added 10^−5^ mol/L CrCl_3_ only decreased the colony numbers slightly, from 100 to 72 CFU per plate.

**Table 1:** The effect of added multivalent cations and cationic surfactants on *Escherichia coli* viability

Added multivalent cations and cationic surfactants	Concentration (mol/L)	Solution pH	Average CFU per plate
Blank group (10^−3^ mol/L Nacl)	—	6.0	100
LaCl_3_	10^−5^	5.6	2
FeCl_3_	10^−5^	3.5	60
AlCl_3_	10^−5^	4.6	50
CrCl_3_	10^−5^	5.2	72
C14TAB	10^−4^	5.8	0
CaCl_2_	0.01	5.2	120
MgCl_2_	0.01	5.3	89
ZnSO_4_	0.01	5.4	80

### 3.3 Dynamic light scattering study of trivalent ion effects on E. coli cells

**Figure 2:**
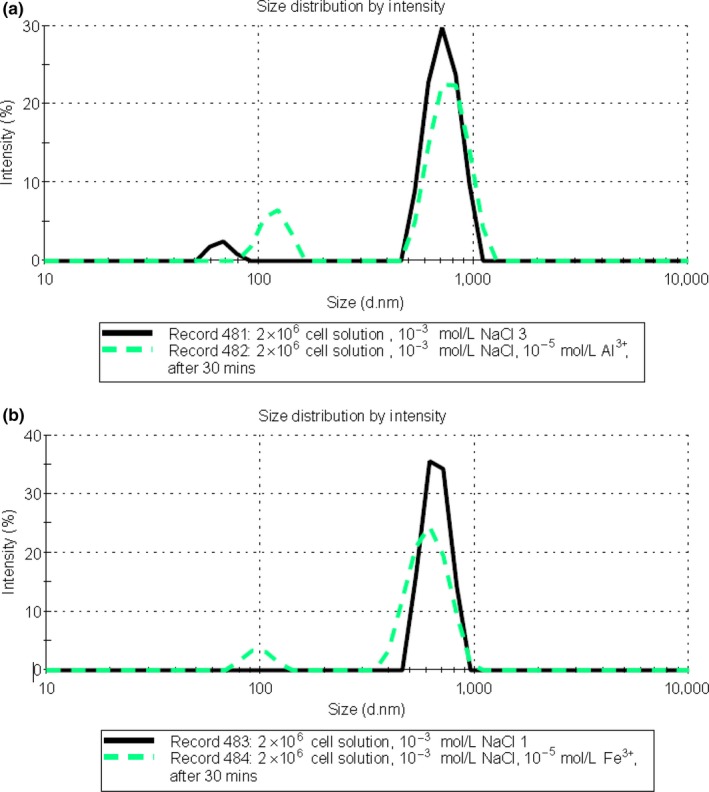
Comparison of particle size distributions measured using DLS at 25°C for 2 × 10^6^ CFU/ml density ATCC 11775 strain *E. coli* sample. (a) in the presence of 1 mmol/L NaCl solution (bold), 10^−3^ mol/L NaCl, and 10^−5^ mol/L AlCl_3_ solution after 30 min (dotted dash line). (b) in the presence of 1 mmol/L NaCl solution (bold), 10^−3^ mol/L NaCl, and 10^−5^ mol/L FeCl_3_ solution after 30 min (dotted dash line)

In order to understand the dramatic effect of La^3+^ ions on *E*. *coli* incubation, DLS was used to monitor aggregation changes and cell size changes. The reports given in Figure 2 illustrated that the addition of Fe^3+^ and Al^3+^ have little or no effect on the *E*. *coli* cell size distribution in terms of the mean peak value. This is most likely due to the fact that the percentage of cell aggregation in the solution by the presence of added Fe^3+^ and Al^3+^ is relatively low. In addition, the size of the aggregates in the solution may not be big enough to clearly distinguish them from the single cells. This observation is quite similar with DLS in protein aggregation studies (Arzensek, Podgornik, & Kuzman, 2010).

**Figure 3:**
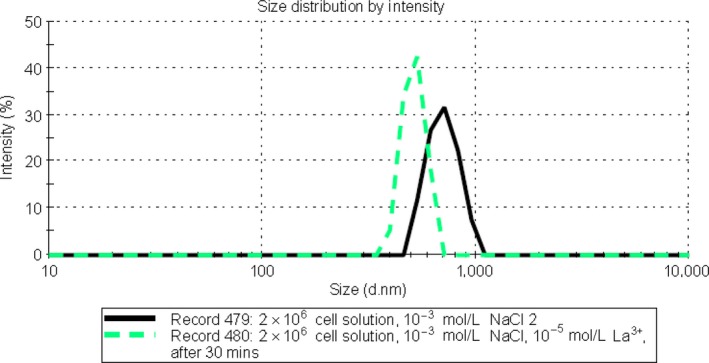
Comparison of particle size distributions measured using DLS at 25°C for 2 × 10^6^ CFU/ml density ATCC 11775 strain *E. coli* in the presence of 10^−3^ mol/L NaCl solution (bold), 10^−3^ mol/L NaCl, and 10^‐5^ mol/L LaCl_3_ solution after 30 min (dash line)

However, a comparison of cell size distribution in the 10^−3^ mol/L NaCl solution with and without lanthanum, given in Figure 3 shows a clear reduction in size in the low concentration lanthanum chloride solution. As 10^−5^ mol/L lanthanum was used in this study, the cation concentration effect on peak size movement should be relatively small in this case. Therefore, this size reduction indicates major membrane disruption followed by substantial leakage of internal cell fluids and organelles, since the osmotic pressure of the solution was much lower than that of the interior of the cells. Such size shrinkage also has been reported in cell size studies in C_14_TAB solutions (Li et al., 2015).

**Figure 4:**
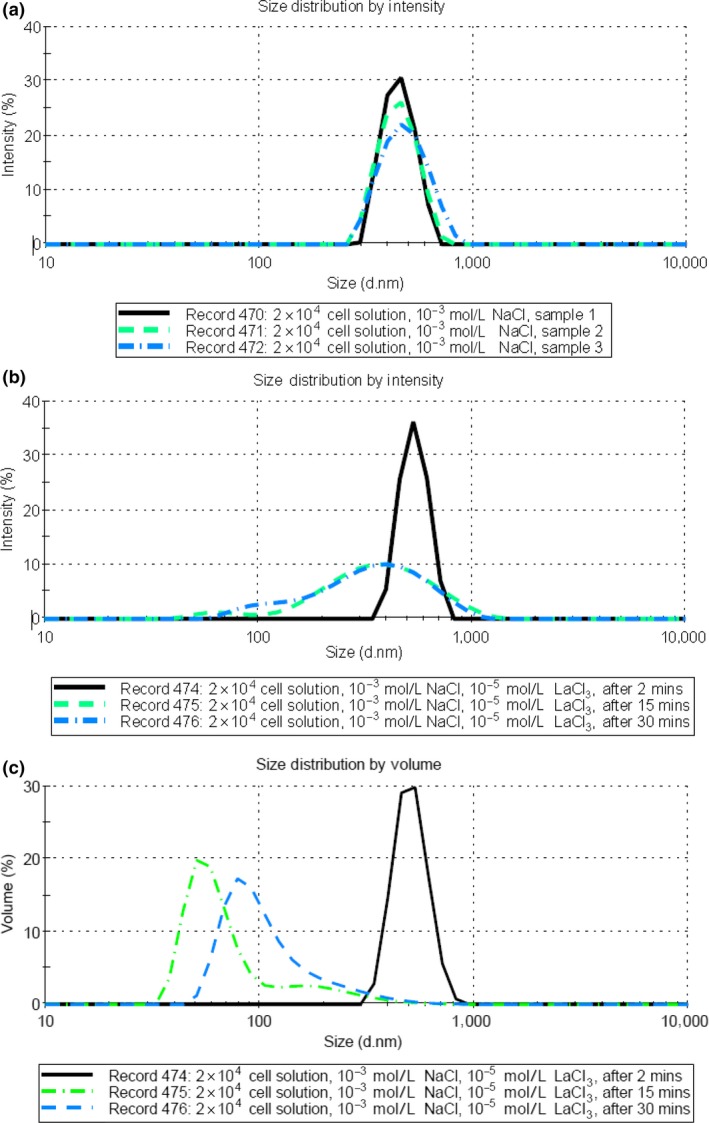
Comparison of particle size distributions using DLS at 25°C for 2 × 10^4^ CFU/ml density ATCC 11775 strain *E. coli* in the presence of different solutions. (a) 2 × 10^4^ CFU/ml density ATCC 11775 strain *E. coli* is in the presence of 10^−3^ mol/L NaCl solution, in the three measurement, the average peak diameter of *E. coli* in this solution is 453 nm and the *Z*‐average is 405 nm. (b) 2 × 10^4^ CFU/ml density ATCC 11775 strain *E. coli* is in the presence of 10^−3^ mol/L NaCl and 10^−5^ mol/L LaCl_3_ solution. The bold line corresponds to the sample after 2 min LaCl_3_ added, the dotted dash line corresponds to the after 15 min well mixture and the dash line corresponds to the after 30 min well mixture (It should be noted that the figure was created by intensity data). (c) 2 × 10^4^ CFU/ml density ATCC 11775 strain *E. coli* is in the presence of 10^−3^ mol/L NaCl and 10^−5^ mol/L LaCl_3_ solution. The bold line corresponds to the sample after 2 min LaCl_3_ added, the dotted dash line corresponds to the after 15 min well mixture and the dash line corresponds to the after 30 min well mixture (It should be noted that The figure was created by volume data)

These effects were also studied in low‐density 2 × 10^4^ CFU/ml *E*. *coli* cell suspensions, as shown in Figure 4. The results obtained from the intensity data in Figure 4b, showed that the substantial signal and significant size reduction occurred in the 10^−5^ mol/L lanthanum chloride solution after 15 min. In the measurements by volume data given in Figure 4c, this size‐shrinking effect was even more clearly demonstrated.

### 3.4 DLS and zeta potential studies of trivalent ion effects on phospholipid vesicles

To further investigate the mechanisms of trivalent ions interaction with the membranes of microorganisms like *E*. *coli*, the size distribution and zeta potential of pure phospholipid vesicles (SUVs) were studied with added Cr^3+^ and La^3+^. These SUVs is close to the composition of typical *E*. *coli* membranes. As mentioned in the introduction, the LPS that contained in the outer membrane of gram‐negative bacteria, like E. coli, contributes to the negative charge of the cell surface through its charged polar groups, such as phosphate and carboxylate groups, which vary in type and number for different bacteria (Kabanov & Prokhorenko, 2010). It was also reported that the surfaces of bacterial gram‐negative cells are identical, containing carboxyl, amide, and phosphate groups, as observed via infrared spectroscopy (Jiang et al., 2004). In addition, a simple membrane surface ionization model (Li et al., 2015), based only on PS and PC components, has been used to model the surface charging properties of *E*. *coli* cells. PS is a typical anionic membrane lipid with phosphate and carboxylate groups, while PC is zwitterionic and has an almost neutral surface contribution (Sou & Tsuchida, 2008). The bilayer of a two‐component vesicle is necessarily slightly asymmetric in order to be thermodynamically stable (Ninham & Nostro, 2010). The chemical groups and surface charging properties of these vesicles closely mirror those of *E*. *coli*, and hence they can reasonably be used to model the interaction effects between typical membranes under the influence of added trivalent cations.

The size distribution obtained for three samples of 0.05 mg/ml SUVs suspended in 0.02 mol/L HEPES/NaOH pH 7.5 buffer and 0.1 mol/L NaCl solution are given in Figure 6. The results are consistent with each other and showed that the SUV has a *Z*‐average size of around 112 nm at 25°C. Mui et al. (2003) has observed a similar size range for PL vesicles. The average zeta potential value of the vesicle was found to be −54.3 mV in the pH 7.5 buffer solution, as shown in the Table 2. Similar zeta potential values (around −50 mV) were also obtained for vesicles with carboxylate anion and phosphate anion (Sou & Tsuchida, 2008).

**Table 2:** Zeta potentials of 0.05 mg/ml PL SUVs in 0.02 mol/L HEPES/NaOH pH7.5 buffer and 0.1 mol/L NaCl (blank group) at 25°C, with and without additive LaCl_3_ and CrCl_3_. The results of the average zeta potentials were obtained from five measurements

Concentration of additive salt (M)	Blank group	LaCl_3_		CrCl_3_	
	0	10^−4^	10^−5^	10^−4^	10^−5^
Average zeta potential (mV)	−54.3	6.5	−25.4	−16.9	−54.1

**Figure 5:**
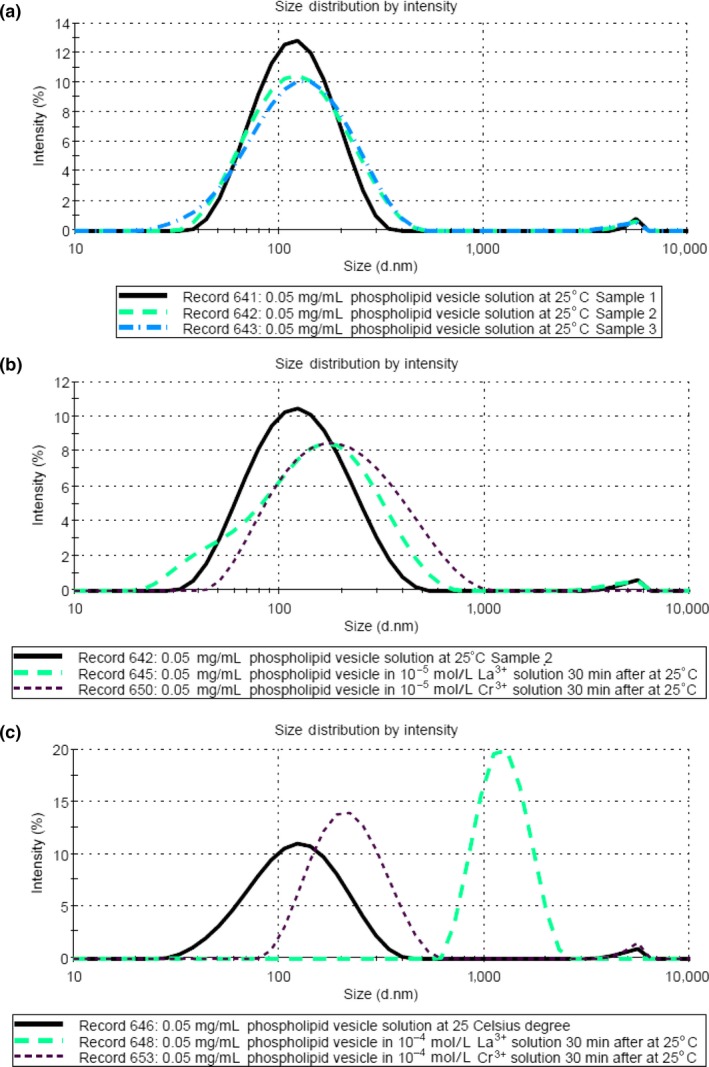
Comparison of particle size distributions by intensity data measured using DLS at 25°C for 0.05 mg/ml phospholipid vesicles in the presence of 0.02 mol/L HEPES/NaOH pH 7.5 buffer and 0.1 mol/L NaCl solution. (a) three lines correspond to the sample without any additive. The measurements show that the *Z*‐average is 112 nm. (b) The bold line corresponds to the sample without any additive, the longer dash line corresponds to the after 10^−5^ mol/L LaCl_3_ added 30‐min well mixture and the shorter dash line corresponds to the after 10^−5^ mol/L CrCl_3_ added 30‐min well mixture. (c) The bold line corresponds to the sample without any additive, the longer dash line corresponds to the after 10^−4^ mol/L LaCl_3_ added 30‐min well mixture, and the shorter dash line corresponds to the after 10^−4^ mol/L CrCl_3_ added 30‐min well mixture

Comparisons of PL vesicle size distribution in different concentrations of Cr^3+^ and La^3+^ solutions at 25°C after 30 min well mixing are shown in Figure 5 (B&C). The corresponding zeta potentials of the PL vesicles in the solutions are also summarized in Table 2. The results given in Figure 5b were obtained using 0.05 mg/ml phospholipid vesicles with 10^−5^ mol/L of LaCl_3_ or CrCl_3_ or without any additive. Both Z‐average sizes measured for added 10^−5^ mol/L LaCl_3_ and CrCl_3_ sample at 25°C slightly increased to a *Z*‐average of about 175 nm, compared with the size in the solution without additive as shown in Figure 5a (*Z*‐average 112 nm). On the other hand, from the zeta potential measurements given in Table 2 10^−5^ mol/L Cr^3+^ ions showed no effect on the membrane surface potential, while the vesicle surface potential increased to −25.4 mV from −54.3 mV for the added 10^−5^ mol/L La^3+^ solution.

On increasing the trivalent ion concentration to 10^−4^ mol/L, the results obtained for Cr^3+^ and La^3+^ (Figure 5c) were quite different. The DLS measurement of the solution with added 10^−4^ mol/L CrCl_3_ behaved in a similar manner to the result with 10^−5^ mol/L CrCl_3_, having a *Z*‐average around 175 nm, but with a higher intensity rate. However, in the 10^−4^ mol/L LaCl_3_ solution, there was a substantial increase in size, from 112 nm to 1200 nm.

## 4 DISCUSSION

### 4.1 Effects of surfactants

Myristyltrimethylammonium bromide (C_14_TAB) molecules are strongly adsorbed into cell membranes even at low concentrations. The significant CFU density reduction might be due to major membrane disruption following substantial leakage of internal cell fluid and organelles, caused by C_14_TAB molecules adsorption into the external membrane, which is consistent with the result summarized in Table 1. It showed that there was a complete decrease in CFU density, even at 10^−4^ mol/L concentration.

The well‐known general detergent action of surfactants occurs around the critical micelle concentration (CMC). The CMC of C_14_TAB is around 3.7 × 10^−3^ mol/L (Lu, Simister, Thomas, & Penfold, 1993) and at that point, the double chained phospholipids of the bilayer membrane are typically solubilized into the micellar aggregates (Mitchell & Ninham, 1981; Ninham & Nostro, 2010). Note, however, that the CMC depends on background salt and charged colloidal aggregates in the suspension (through the Debye length). This process is universal. Such detergent action occurs most effectively for cationic surfactants – different mechanisms of mixing membrane phospholipids and nonionic or anionic surfactants inhibit accomplished membrane destruction. So the positive charge and hydration of the surfactant plays an important and more complex role.

### 4.2 Effects of trivalent cations on *E*. *coli* viability

In addition, Cr^3+^ and La^3+^ ions have very different hydration properties. These differences have been demonstrated in force measurements between mica surfaces. La^3+^ ions appeared to bridge facing mica surfaces creating strong final contact adhesion, whereas Cr^3+^ ions were easily displaced from the mica surfaces before the final contact (Pashley, 1984). These differences were apparently due to the stronger binding of water molecules to the Cr^3+^ ion. So it was assumed that the mechanism of La^3+^ toxicity observed in Figure 1 is similar to that with C_14_TAB, and is more likely due to the weak hydration of the more labile La^3+^ ions.

Depending on Debye length, a function of concentration and background salt, the ionic hydration can allow weakly associated multiply charged ionic aggregates to form. Such aggregates would most likely form on adsorption at the bacterial surface. These might mimic the same self‐assembled associations of cationic surfactants.

Thus, multiple charge ions can associate with the phospholipid membranes, and cause changes in membrane curvature and integrity. Ultimately this posits membrane disruption to compatibility of hydrocarbon chains (CTAB vs. lipids) on one hand and compatibility of head group hydration of lanthanum ions and hydration of the lipid head group in the other.

### 4.3 Effects of trivalent cations on phospholipid membranes

In this study, ion concentrations in the solutions, $C_{i}\left(x\right)$ *,* as a function of distance *x* from the vesicle charged surface, which can be considered as a flat surface with respect the ion diffuse double layer, can be described by the Boltzmann distribution function (Pashley & Karaman, 2005), that is:

(1) $$C_{i}\left(x\right)=C_{i}\left(b\right)\exp\left[\frac{-z_{i}\left|q_{e}\right|\psi_{x}}{\mathrm{kT}}\right]$$

where $C_{i}\left(b\right)$ is the bulk concentration of ion “*i*”, *z*_*i*_ is the valency of the ion, and $\psi_{x}$ is the electrostatic potential at distance *x* from the flat surface. *q*_*e*_ is the electron charge and *k* is the Boltzmann constant. *T* is the absolute temperature (in *K*).

Although the concentrations of added LaCl_3_ or CrCl_3_ were much lower than 0.1 mol/L NaCl in the solutions, La^3+^ or Cr^3+^ ions with a significant higher valency (*z*_*i*_) than Na^+^ would be more highly concentrated near to the negatively charged surfaces, as predicted from Equation (1). In other words, the trivalent ions even in this low concentration could effectively screen the repulsive electrostatic force acting between the negatively charged phospholipid headgroups. This effect will serve to reduce the repulsion and hence the lipid head group area. Molecular organization of lipids in self‐assembled structures, such as membranes, depends upon a range of competing intramolecular forces, the flexibility of the chains and intermolecular forces (Pashley & Karaman, 2005). These forces all influence the lipid aggregate architecture and stability and the prediction of the physical features of this kind of aggregation seems to be complicated.

However, simple packing constraints based on the degree of curvature existing at the aggregate surface offers a valuable tool to define the geometry of self‐assembled microstructures. This curvature can be expressed as a parameter known as the critical packing parameter, $v_{H}/\left(\alpha_{P}l_{H}\right)$ *,* where $v_{H}$ and $l_{H}$ are the hydrocarbon chain's volume and length, and α_P_ is the cross‐section area of the polar headgroup (Ninham & Nostro, 2010; Pashley & Karaman, 2005; Tanford, 1973). For example, when $v_{H}/\left(\alpha_{P}l_{H}\right)$ >0.5 and <1, structures such as single‐walled vesicles or liposomes would be formed (Ninham & Nostro, 2010).

Hence, a reduction in electrical repulsive force between the charged headgroups, due to the presence of trivalent cations in solution, was expected to increase the value of the critical packing parameter and so reduce the curvature of the vesicles, which is supported by the observed increase in vesicle size (see Figure 5c). On further increasing the trivalent ion concentration to 10^−4^ mol/L, these results suggested that La^3+^ ions can cause intervesicle binding but not Cr^3+^ ions, under similar solution conditions.

In the experimental results (Table. 2), La^3+^ and Cr^3+^ in the lower concentration level (10^−5^ mol/L) have the similar effect on the PL size distribution, but have a different effect on the zeta potential. This is might be due to their very different hydration properties as mentioned before. By comparison, hydrated La^3+^ ions are labile allowing rapid hydration water exchange; hence, hydration water can be replaced by other polar groups on the vesicle surface, so that the ions could bind strongly with the negatively charged surface. This is supported by the zeta potential measurements in lower level 10^−5^ mol/L La^3+^ and Cr^3+^ effects study in Table 2, which showed that adding La^3+^ caused the zeta potential value to increase from −54.3 mV to −25.4 mV, while this level of Cr^3+^ did not change the zeta potential value.

**Figure 6:**
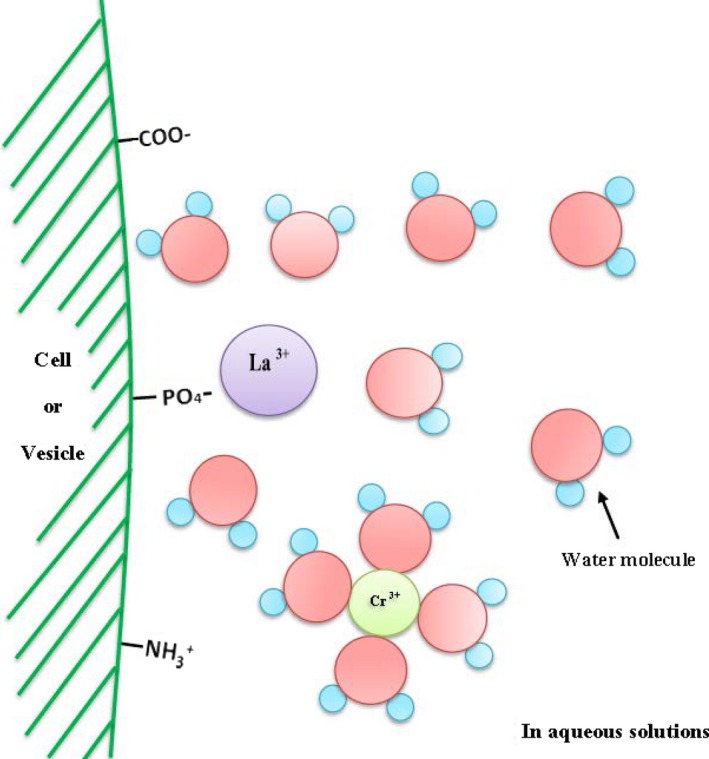
Schematic *E. coli* cell or vesicle surface in the presence of low levels of trivalent cations solutions

Both Cr^3+^ and La^3+^ can be considered as hydrated $M\;{\left(\mathrm{OH}\right)}_{6}^{3+}$ ions, as shown in the schematic diagram Figure 6, but it has been established that the water molecules coordinated around the Cr^3+^ are more strongly bound and their exchange rate with bulk water very slow due to the intermediate state required for exchange being very unfavorable (Pashley, 1984). Hence, when the lipid surface adsorbs the Cr $\text{CR}{\left(\mathrm{OH}\right)}_{6}^{3+}$ ion, it is unlikely to lose any of its coordinated water molecules.

Therefore, the added higher level (10^−4^ mol/L) of CrCl_3_ in the solutions might only vary the local surface electrical field, rather than have an influence on vesicle aggregation. As we observed, there was actually no effect on the size by increase in the concentration of CrCl_3_, as shown in Figure 5c, while the vesicle surface potential, reported in Table 2, did increase to −16.9 mV from −54.3 mV.

On the other hand, adding 10^−4^ mol/L La^3+^ caused the zeta potential value to increase from −54.3 mV to + 6.5 mV. It seems that in this cationic environment, the vesicles become thermodynamically unstable and so form aggregates. A similar result for small PS vesicles was found by Hammoudah et al., (1979) who reported that the trivalent La^3+^ ion could cause significant local rigidity, inducing aggregation, fusion, and leakage of the small PS vesicles at a small binding ratio. Based on these findings, it can be assumed that LPS has similar charged groups to PS in the *E*. *coli* cell membrane and so might behave in a similar manner in the presence of added La^3+^ ions. The strong effect on the LPS and PS components due to La^3+^ adsorption is probably a critical factor in causing the *E*. *coli* cells outer and inner membrane disruption, leading to cell size shrinkage, as was found in Figure 5, and caused cell inactivation during incubation, as illustrated in Figure 1.

## CONFLICT OF INTEREST

The authors declare that they have no competing interests.
